# A76 MODULATING THE PREOPERATIVE GUT MICROBIOTA USING DIETARY FIBER TO IMPROVE COLORECTAL CANCER SURGICAL OUTCOMES

**DOI:** 10.1093/jcag/gwae059.076

**Published:** 2025-02-10

**Authors:** C McCartney, G Fragoso, A Calve, C Gerkins, T Cuisiniere, A S Ajayi, A Alaoui, R Hajjar, N Taleb, C Richard, M M Santos

**Affiliations:** Centre de Recherche du Centre Hospitalier de l’Universite de Montreal, Montreal, QC, Canada; Centre de Recherche du Centre Hospitalier de l’Universite de Montreal, Montreal, QC, Canada; Centre de Recherche du Centre Hospitalier de l’Universite de Montreal, Montreal, QC, Canada; Centre de Recherche du Centre Hospitalier de l’Universite de Montreal, Montreal, QC, Canada; Centre de Recherche du Centre Hospitalier de l’Universite de Montreal, Montreal, QC, Canada; Centre de Recherche du Centre Hospitalier de l’Universite de Montreal, Montreal, QC, Canada; Centre Hospitalier de l’Universite de Montreal, Montreal, QC, Canada; Centre Hospitalier de l’Universite de Montreal, Montreal, QC, Canada; Centre Hospitalier de l’Universite de Montreal, Montreal, QC, Canada; Centre Hospitalier de l’Universite de Montreal, Montreal, QC, Canada; Centre de Recherche du Centre Hospitalier de l’Universite de Montreal, Montreal, QC, Canada

## Abstract

**Background:**

Anastomotic leak (AL) is a postoperative complication that occurs in up to 20% of patients undergoing surgery for colorectal cancer (CRC). It is characterized by the poor healing of the intestinal reconnection and is associated with increased mortality, morbidity, and cancer recurrence. The gut microbiota plays a key role in anastomotic healing, potentially mediated by the production of beneficial short-chain fatty acids (SCFA). Supplementation with the dietary fiber inulin was shown to increase SCFA as well as improve microscopic and macroscopic anastomotic healing in a mouse surgical model. However, when considering clinical applications, differences in baseline microbiota composition and patient ability to respond to a dietary fiber must be taken into account.

**Aims:**

In order to differentiate between responders and non-responders prior to surgery, we tested patient responses to different fibers in a mouse fecal microbiota transplantation (FMT) model. Our overall objective is to identify microbial or systemic markers that could predict patient response to different fibers, allowing for personalized dietary interventions.

**Methods:**

Wild-type C57BL/6 mice received FMT from a human donor. Following a 2 week engraftment period, mice received supplementation with one of four dietary fibers for 2 weeks, after which fecal samples were collected for SCFA analysis using HPLC-MS.

**Results:**

The microbiota response to each dietary fiber was estimated based on increased fecal SCFA levels at endpoint for each FMT donor.

**Conclusions:**

Our FMT mouse model is able to detect increased SCFA levels in response to dietary fiber supplementation. Future validation will include measuring post-operative intestinal healing parameters in a mouse surgical model and comparing these results with an ongoing clinical trial. By lowering the risk of AL, we aim to decrease treatment burden for CRC patients and improve their quality of life post treatment.

**Funding Agencies:**

CIHRFRQS

